# Population attributable fractions of psychiatric disorders for suicide in low and middle-income countries: a systematic review and meta-analysis

**DOI:** 10.1186/s12888-026-08252-1

**Published:** 2026-06-03

**Authors:** S. M. Yasir Arafat, Vikas Arya, Srijony Ahmed, Sujita Kumar Kar, Vikas Menon, Murad M. Khan, Matthew J. Spittal, Duleeka Knipe

**Affiliations:** 1 Department of Psychiatry, Bangladesh Specialized Hospital, Dhaka, 1207 Bangladesh; 2 Department of Public and Community Health, Faculty of Medicine and Health Sciences, Frontier University Garowe, Puntland, Somalia; 3 Melbourne School of Population and Global Health, The University of Melbourne, Melbourne, Australia; 4 Department of Psychiatry, Dhaka Community Medical College and Hospital, Dhaka, Bangladesh; 5 Department of Psychiatry, King George’s Medical University, Lucknow, UP 226003 India; 6 Department of Psychiatry, Jawaharlal Institute of Postgraduate Medical Education and Research, Puducherry, 605006 India; 7 Brain & Mind Institute, Aga Khan University, Karachi, 74000 Pakistan; 8 Population Health Sciences, Bristol Medical School, University of Bristol, Bristol, UK; 9 South Asian Clinical Toxicology Research Collaboration, Faculty of Medicine, University of Peradeniya, Peradeniya, Sri Lanka

**Keywords:** Psychiatric disorder, Population attributable fractions, Low-and middle income country, Suicide, Depression

## Abstract

**Background:**

Low and middle-income countries (LMICs) have a high burden of suicide with limited resources. The population attributable fraction (PAF) is a valuable tool to help policymakers prioritise suicide prevention activities.

**Objectives:**

To estimate the PAFs of psychiatric morbidity in suicide in LMICs.

**Methods:**

We searched MEDLINE, Embase, and PsycINFO for empirical studies assessing the prevalence of psychiatric disorders among suicide in LMICs. This is an update and extension of a previous review. We meta-analysed estimates of associations, and calculated PAFs with 95% confidence intervals (CI) for different psychiatric disorders in relation to suicide. Publication bias was assessed using contour plots.

**Results:**

14 studies which met our eligibility criteria were included. Five studies were published from China, 13 were case-control studies, and all the studies gathered data between 2000 and 2021. The studies assessed 2132 suicides. Estimates from 10 reasonable quality studies contributed to our meta-analysis. The PAF was 62.1% (95% CI 48.5%-73.6%) for any psychiatric disorder, 28.4% (12.7%-50.1%) for mood disorder, and 23.0% (9.7%-42.8%) for depression. There was substantial heterogeneity in association estimates for these exposures between included studies. PAFs ranged between 1.6% and 7.6% for all other psychiatric disorder categories identified. Contour plots indicated publication bias.

**Conclusions:**

Treating psychiatric disorders could reduce the incidence of suicide by more than a half in some LMICs. Our findings need to be interpreted with caution due to data not being available from 94% of LMICs and evidence of publication bias which would lead to overestimations in PAF estimates.

**Supplementary Information:**

The online version contains supplementary material available at 10.1186/s12888-026-08252-1.

## Introduction

Suicide is a global public health problem causing more than 727,000 deaths annually. Most of these deaths (73%) occur in low and middle-income countries (LMICs) [1]. Psychiatric disorders have been identified as an important risk factor for suicide in many countries, even though there is substantial variation in rates between countries and regions [2–5]. Of the psychiatric disorders, mood disorders and substance use disorders are the most prevalent conditions associated with suicide [6].

Despite LMICs experiencing the greatest burden of suicide globally, there is a dearth of good quality research, and suicide prevention is not prioritised [7, 8]. Whilst psychiatric disorders have been identified as being important precursors to suicide in many high-income countries, this association is less apparent across LMICs [2]. In contexts where economic and health-care resources are limited at national levels for suicide prevention, understanding and prioritising the most effective targets for prevention is important. In this study, we aimed to determine the population attributable fraction (PAF) for psychiatric morbidity among suicide deaths in LMICs. Assuming a causal relationship, a PAF gives a measure of how much of a health outcome could be prevented if a particular exposure is eliminated from the population. It is therefore a useful public health tool to help countries to prioritize resources for suicide prevention, especially in countries where they are formulating national suicide prevention strategies and policies.

## Methods

We followed the PRISMA guidelines and registered the review protocol on PROSPERO (CRD42021287416).

### Search strategy and selection criteria

This review is linked to an earlier review (PROSPERO 2018 CRD42018087851) [2]. We used the same search criteria as the previous review which covered 1st January 1990 to 25 February 2018. In this study, we provide an update of the evidence by conducting searches to include all new papers published between 26 February 2018 and 10 July 2023. This review, therefore, included eligible research published between 1 January 1990 to 10 July 2023.

The search was conducted on the 10th July 2023 in MEDLINE, Embase, and PsycINFO. The search strategy is available in supplementary methods. We also searched the reference lists of eligible papers and conducted citation searches of key papers to identify additional eligible reports. Identified titles and abstracts were screened using Covidence for eligibility for full text review in the same software.

We restricted this review to populations in LMICs as per the World Bank’s classification (http://data.worldbank.org/about/country-classifications). No language, age, sex, or other demographic group restrictions were applied in the search. We restricted the included study designs to case-control and cohort studies. This was to allow us to have an estimate of the population level of psychiatric disorders within the same study population – an estimate which is needed in order to calculate PAF. Only studies that used a semi-structured or structured instrument to measure psychiatric exposures were included. Studies were included if we could extract counts of the following: (1) the number who died by suicide and were exposed; (2) the number who died and were *not* exposed; (3) the number who did *not* die by suicide and were exposed; and (4) the number who did *not* die by suicide and were *not* exposed.

We intended to identify reliable and objective estimates among the general population. Therefore, studies conducted in clinical or special populations (e.g., obese individuals, veterans, psychiatric patients) were excluded. We excluded studies where no structured or semi-structured instrument was used to ascertain the exposures, or if it was unclear that a structured or semi-structured instrument had been used.

### Data extraction

The following data were extracted using a pre-piloted data extraction sheet from each study: study setting; data collection duration; study design (case-control or cohort study); data collection method; name of structured instrument used for diagnosing exposure to psychiatric disorders; type of disorder (any psychiatric disorder, any axis-1 disorder, depression disorder, substance use disorder, personality disorder, psychosis, stress disorder, bipolar disorder, anxiety disorder, mood disorder, psychotic disorder, schizophrenia-spectrum disorder, sleep disorder), the counts of the number of people who died and did not die of suicide and the number exposure and unexposed to the disorders, corresponding to the four groups described above. Data were extracted independently for each paper by two researchers.

### Quality assessment

The overall quality of each study was evaluated and rated according to the *Joanna Briggs Institute* (JBI) critical appraisal checklist for case-control and cohort studies. We assessed the study quality against the mentioned criteria of the checklist. A study was considered to be of reasonable quality (i.e. at a lower risk of bias) if they used a structured instrument (e.g. SCID) of psychiatric disorder. Studies which used other methods such as record reviews were considered to be at a higher risk of bias given the limited mental health services within most LMICs which means that a large number of people who experience psychiatric disorders are unlikely to be formally diagnosed. The quality was assessed by two review team members (VA and DK) independently. We present the rating using web app *robvis* [9].

### Statistical analysis

We report descriptive information about the included studies. We estimated the PAF for each psychiatric exposure where there were at least three studies available for pooling. We only included studies in our primary meta-analysis if they were of reasonable quality. This was done to reduce the degree of heterogeneity between estimates. In our protocol we specified that we would provide estimates by region, but given the relatively few studies identified in this review we were only able to provide overall estimates. In order to synthesis estimates we grouped disorders together if they were broadly similar, e.g. Axis I, SCID-I and any psychiatric disorder into one group. As the PAF is derived from two different estimates - the proportion of the population exposed and the odds ratio of the association between the exposure and the outcome – our meta-analysis was undertaken in three stages. First, for each exposure we estimated the mean proportion of the population exposed ($\:{P}_{e})$. For case-control studies, we calculated this using only the proportion exposed in the control group (the non-cases of suicide). For cohort studies, this was calculated using the total cohort [10]. Second, we used a random effects meta-analysis to estimate the pooled odds ratio (* OR*) that represents the association between each exposure and the outcome. When calculating the OR and their standard errors prior to pooling, we added 0.5 to all cells if one or more cells had a value of zero. Finally, we calculated the PAF, where the PAF was defined as:

$$\:\mathrm{PAF}=\frac{{P}_{e}\times\:\:(OR-1)}{1+{P}_{e}\times\:\:(OR-1)}$$

We also estimated the upper and lower limits of the PAF, which were derived by recalculating the PAF using the upper and lower 95% confidence interval limits of the pooled OR. Heterogeneity was assessed using the I^2^ statistic with source of large I^2^ values investigated using meta-regression with an indicator variable representing how cases were identified (hospital records vs. other source). Publication bias was assessed using contour enhanced funnel plots. We undertook a sensitivity analysis calculating the PAFs using all available studies (i.e. irrespective of the quality rating). All analyses were undertaken in Stata version 19.0.

### Human ethics and consent to participate declarations

Not applicable.

## Results

**Fig. 1:**
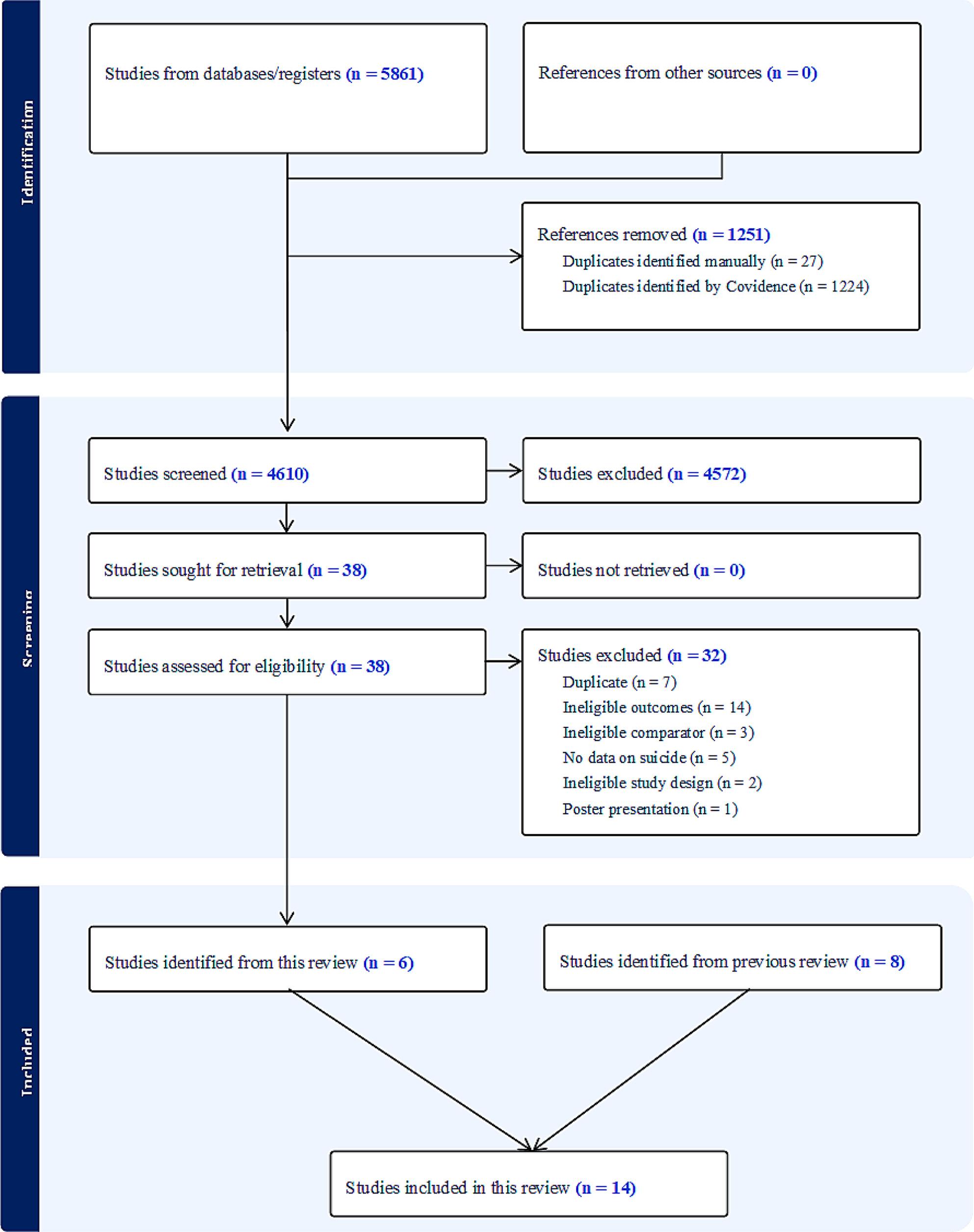
PRISMA 2020 flow diagram

**Fig. 2:**
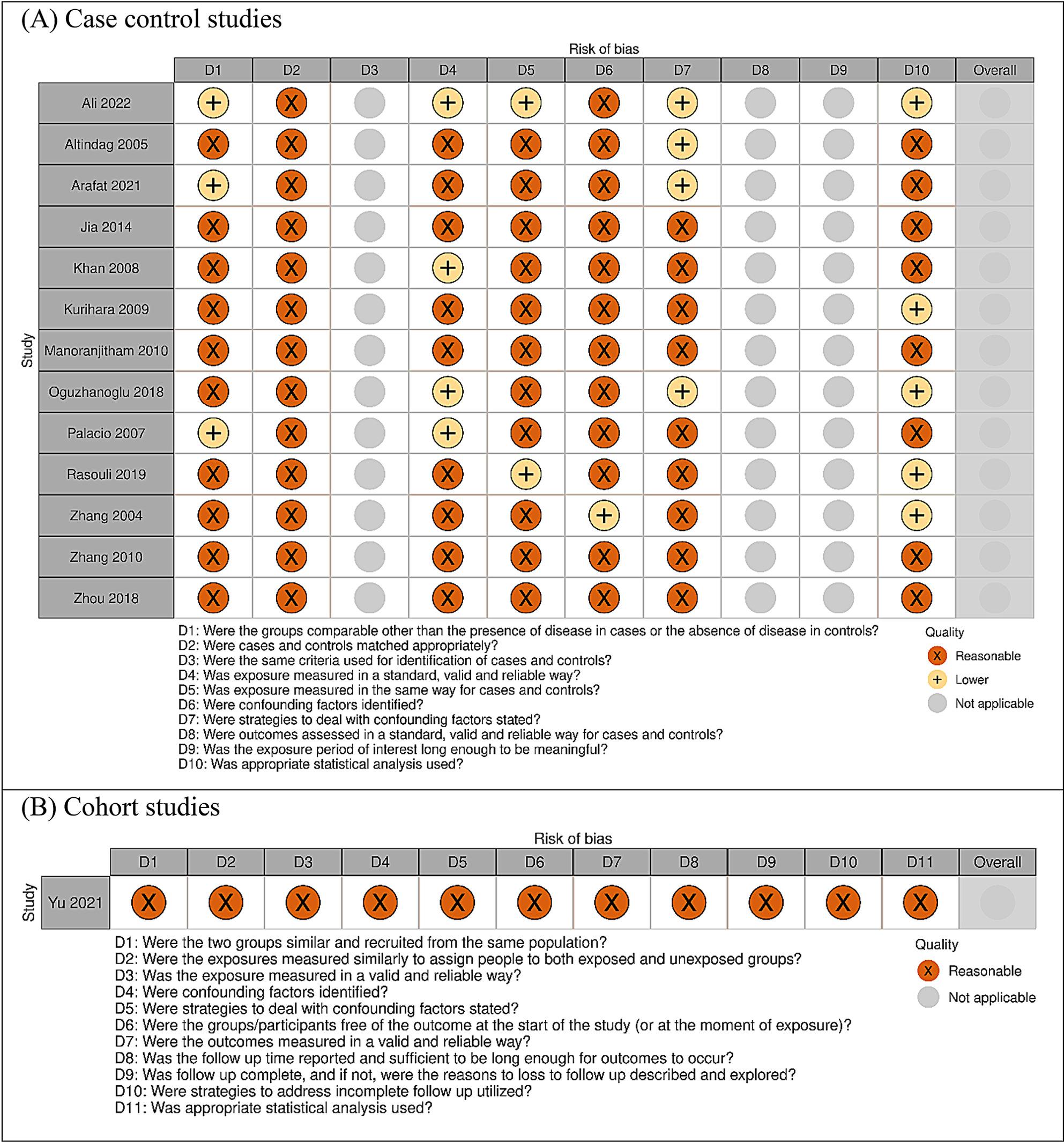
Quality assessment of included studies

Eight articles from the previous review were eligible for inclusion in our current study [2]. The detailed stepwise process of our updated search is outlined in Fig. 1. In total, fourteen studies were included [11–24], with 10 rated as reasonable quality (Table 1 and Fig. 2). There were five studies conducted in China, two each from Türkiye and Pakistan, and one study each from Bangladesh, Colombia, Indonesia, India, and Iran (Table 1). Across these fourteen studies, we analysed data on 2132 suicides. We found no eligible studies from 119 of the 127 LMICs (94%).

**Table 1:** Characteristics of included studies

Study	Country	Data collection duration	Study design	Data collection method	Source of cases	Source of controls	Sample size	Number of cases
Ali 2021 [11]	Pakistan	2018–2021	Case-control	Psychological autopsy	Cases were identified from hospital medical records in Punjab, Pakistan	Living controls were next of kin, relatives, friends or neighbours of the deceased	200	100
Altindag 2005 [12]*	Türkiye	2000	Case-control	Psychological autopsy	Cases were identified from public prosecutor’s records in Batman, Türkiye	Living controls were selected from general practitioner records matched for age, gender and marital status	51	26
Arafat 2021 [13]*	Bangladesh	2019–2020	Case-control	Psychological autopsy	Cases were identified from data held by two medical colleges in Dhaka, Bangladesh	Living controls residing in close vicinity to the cases, matched by sex and age	200	100
Jia 2014 [14]*	China	2008–2009	Case-control	Psychological autopsy	Cases were identified from mortality records in 25 townships in the Shandong Province	Living controls from same township matched by sex and age	400	200
Khan 2008 [15]	Pakistan	2003	Case-control	Psychological autopsy	Cases were identified from police records in Karachi	Living controls from the same area matched by sex and age	200	100
Kurihara 2009 [16]*	Indonesia	2007	Case-control	Psychological autopsy	Cases were identified from police records in Bali	Living controls from the same village matched by sex and age	180	60
Manoranjitham 2010 [17]*	India	2006–2008	Case-control	Psychological autopsy	Cases were identified from a health surveillance system in the Vellore District, Tamil Nadu	Living controls from the same area matched by sex and age	200	100
Oguzhanoglu 2018 [18]	Türkiye	2009–2010	Case-control	Psychological autopsy	Cases were identified from hospital medical records in Denizli, Türkiye	Living controls from primary health care centres from similar location and social background	84	53
Palacio 2007 [19]	Colombia	unknown	Case-control	Psychological autopsy	Cases were identified from forensic records in Medellín, Colombia	Deceased controls who died from accidental injuries; drawn from the same hospital	216	108
Rasouli 2019 [20]*	Iran	2017	Case-control	Psychological autopsy	Cases were identified from hospital medical records in Tehran.	Living controls from the same area matched by sex and age.	80	40
Yu 2021 [21]*	China	2004–2016	Prospective Cohort	Baseline interview then participants followed up through the official disease surveillance system	China Kadoorie Biobank	N/A	512,161	520
Zhang 2004 [22]*	China	2001–2002	Case-control	Psychological autopsy	In one district, cases were identified from records held by the local public health agency. In the other district, cases were identified by interviewing villagers in two townships.	Living controls from the same area matched by sex and age	132	66
Zhang 2010 [23]*	China	2005–2008	Case-control	Psychological autopsy	Multistage sampling was used to first select 12 counties from 3 providences. Cases in these counties were then identified from death certification system.	Living controls living in the same area matched for age and gender	808	392
Zhou 2018 [24]*	China	2014–2015	Case-control	Psychological autopsy	Multistage sampling was used to first select 12 counties from 3 providences. Cases in these counties were then identified from death certification system.	Living controls from the same area matched by sex and age	484	242

*Reasonable quality

All the included studies, except one [21], followed a case-control study design with data from cases collected using the psychological autopsy method and living controls sourced from the same area and matched by age and sex (Table 1). The remaining study utilised data from a prospective cohort, the China Kadoorie Biobank. The studies were published between 2005 and 2021 with data collected between 2000 and 2021. The case-control studies generally used 1:1 matching with cases ranging from 40 suicides to 392 suicides. A total of 520 suicides were observed in the cohort study.

**Fig. 3:**
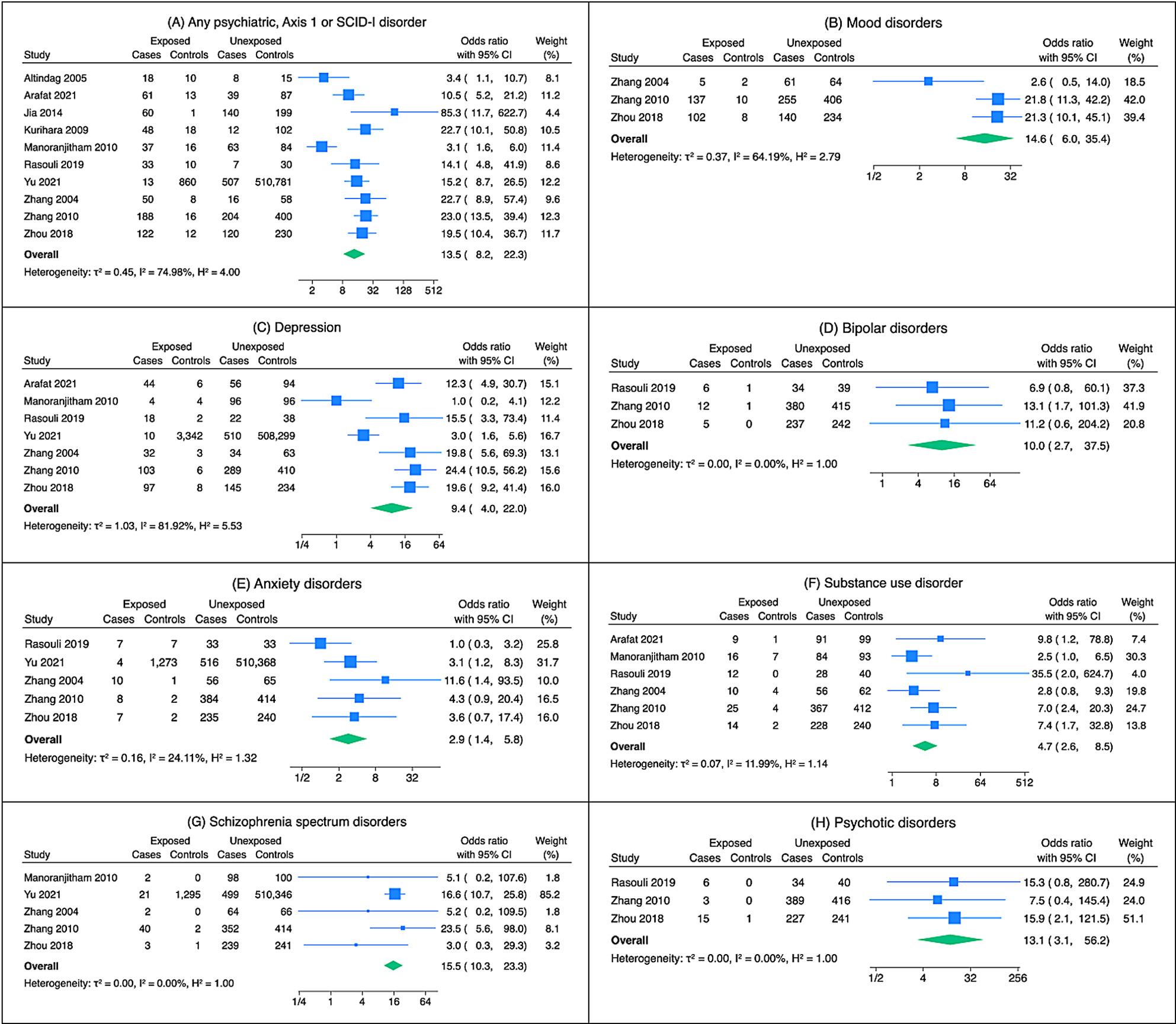
Forest plots of odds of suicide by exposure to psychiatric disorders

Studies that were rated as reasonable quality reported on a range of psychiatric disorders, with a total of 11 categories of disorder reported. Sleep and personality disorders had only 1 estimate each [13, 21], and stress disorders had 2 [17, 23]. For our primary analyses, there were sufficient studies to pool the data for eight psychiatric exposures: any psychiatric (including studies looking at axis 1 or SCID-I disorders only, 10 studies), depression (7 studies), schizophrenia-spectrum disorders (5 studies), anxiety (5 studies), psychotic disorders (3 studies), mood disorders (3 studies), bipolar (3 studies) and substance use (6 studies) (Fig. 3; Table 2). The pooled ORs ranged between 2.9 (anxiety) and 15.5 (schizophrenia-spectrum disorders), with a substantial heterogeneity observed between study estimates for any psychiatric disorder, mood disorders, and depression. There was negligible heterogeneity between study estimates for the other psychiatric exposures. The PAF point estimate was greatest for any psychiatric disorder (62.1% 95% CI 48.5%-73.6%), and lowest for psychotic disorders (1.6% 95% CI 0.3%-7.1%) (Table 2). Heterogeneity was high for depression (82%), any psychiatric disorder (75%) and mood disorders (64%); moderate for anxiety disorders (24%) and substance use disorders (12%); and low for bipolar disorders, schizophrenia spectrum disorders and psychotic disorders (all 0%). Meta-regression indicated no meaningful change in these I^2^ values when the source of the cases was controlled for.

**Table 2:** Population attributable fractions for exposure to psychiatric disorders and suicide

Exposure	Mean proportion exposed	PAF (95% CI)
Any psychiatric, axis 1 or SCID-I disorder	13.1%	62.1% (48.5%-73.6%)
Mood disorders	2.9%	28.4% (12.7%-50.1%)
Depression	3.6%	23.0% (9.7%-42.8%)
Substance use disorder	2.6%	9.0% (4.1%-16.5%)
Bipolar disorders	0.9%	7.6% (1.5%-25.0%)
Anxiety disorders	4.1%	7.1% (1.7%-16.5%)
Schizophrenia spectrum disorders	0.2%	3.2% (2.1%-4.9%)
Psychotic disorders	0.1%	1.6% (0.3%-7.1%)

**Fig. 4:**
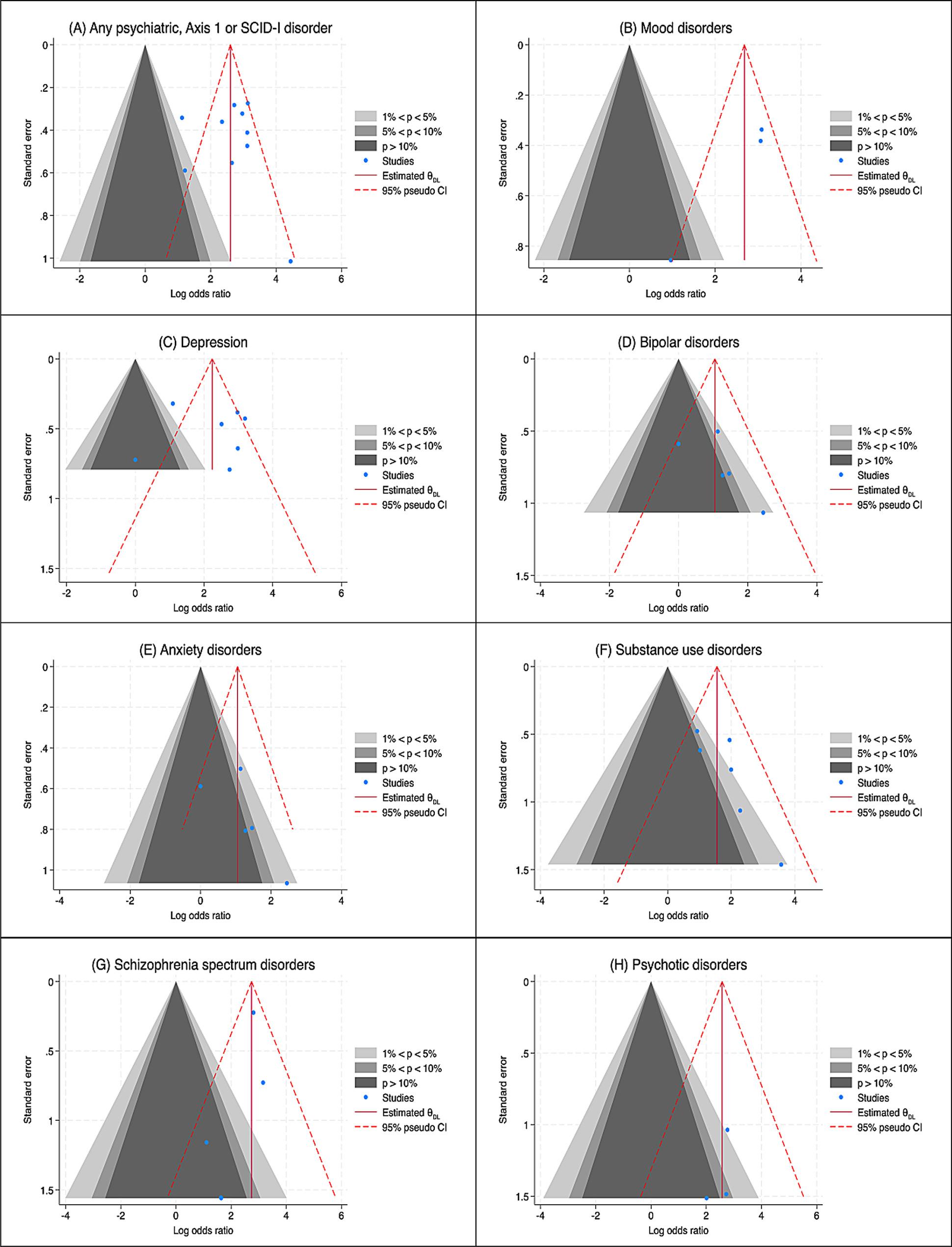
Contour enhanced funnel plots for psychiatric exposures

The contour enhanced funnel plots (Fig. 4) suggest potential publication bias. For all exposures, the studies were mostly within the pseudo 95% confidence intervals. Few studies were within the region representing *p* > 5%, and scarcely any in the region representing *p* > 10%, indicating that studies with null findings may not have been published.

In sensitivity analyses using all studies, PAF point estimates were similar, ranging from 67.3% (95% CI 54.9%-77.4%) for any psychiatry disorder to 1.1% (95% CI 0.2%-4.3%) for psychotic disorders (see supplement Table S1).

## Discussion

Though the relationship between psychiatric morbidity and suicide is well documented in high-income countries, our study adds to the discussion by providing quantitative estimates of the burden of suicide in the general population that is attributable to psychiatric disorders in LMICs. We calculate a PAF which suggests that, assuming a causal relationship, if psychiatric disorders were effectively managed then 62.1% (95% CI 48.5%-73.6%) of suicides could be prevented in LMICs. Among psychiatric disorders, mood disorders and depression had the largest PAF of 28.4% (12.7%-50.1%) and 23.0% (9.7%-42.8%) for suicide. The PAFs for the other disorders that we could estimate were lower and ranged between 1.6% for psychotic disorders to 9.0% for substance use disorders.

Globally, mental disorders remain a major risk factor for suicide. A meta-analysis of the prevalence of mental disorder in suicide found the pooled prevalence of any disorder among suicides to be 81% (95% CI 76%-85%) [4], this is higher than a more recent review which focused on middle-aged adults which reported a prevalence of 58% (95% CI 47%-68%) [25]. The estimates in both reviews were driven by estimated from HICs. A recent LMIC-focussed review yielded a 58% (95% CI 46%-71%) pooled prevalence rate for psychiatric disorders among those who died by suicide [2]. A previous review of studies from HICs [26] estimated the attributable risk of affective disorders, substance use, and anxiety disorders to be 26% (95% CI 7-45%), 9% (95% CI 5-24%), and 5% (95% CI 1%-10%) among males; the corresponding estimates for females were 32% (95% CI 19-67%), 25% (95% CI 5-32%), and 12% (95% CI 6-22%). Though these PAF rankings for these psychiatric disorders are broadly consistent with what we found for mood disorders, depression, substance use, and anxiety disorders, the magnitudes of some individual estimates differ. For example, the PAF point estimates for substance use disorders in our review are at the lower end (PAF 4.8 95% CI 1.6–11.8) compared to previous estimates. Nevertheless, the estimates for depression/mood and anxiety disorders are consistent, suggesting that the effective treatment and eradication of these disorders can lead to a reduction in suicide by roughly a quarter. Possible approaches for intervention in LMICs where formal mental health provision is lacking might be through lay community counsellors [27]. Caution, however, is advised before investment in such approaches, as our review highlights that studies which failed to report statistical evidence of an association between a psychiatric exposure and suicide were less likely to be published. This might be because either an association between psychiatric morbidity and suicide was absent, or more likely the studies were smaller in size and therefore not powered to be able to detect anything but very large effects. The knock-on effect of this publication bias is that our PAF estimates are likely to be higher than would be expected if we were able to identify and include all relevant evidence.

Our findings must be interpreted keeping in mind the limitations of the included studies. The included studies were restricted to just 8 countries and largely included only small regions within these countries. This indicates a pressing need for more studies from other regions where little data are currently available to draw conclusions that are more reflective of the global scenario. We also acknowledge heterogeneity in study setting and populations: studies were conducted in both rural and urban populations as well as using large administrative databases. In part, this may be reflected in the heterogeneity observed in study estimates of PAF between study regions and any/individual psychiatric diagnoses such as depression. Given this scenario, there is a pressing need for more methodologically strong research, particularly from low resource countries to generate more information that will inform and balance resource allocation for preventive efforts. Finally, most studies used a psychological autopsy design with living controls. This approach has been criticised for its high risk of bias. Nonetheless, it represents the best available data in which to estimate the burden of psychiatric diseases on suicide in these settings [28].

## Implications

Our estimates for PAFs of individual psychiatric disorders are mostly consistent with those reported in mostly high-income countries, but it is important to note that very few estimates informed our analysis. The paucity of evidence from many LMICs needs to be taken into account when considering the implications of this study. We know that suicide rates differ by regions, and evidence indicates that heterogeneity in the prevalence of psychiatric disorders in suicide and self-harm differs by LMIC region and that the behaviour is likely to be different [2, 29, 30]. The findings of this review therefore are unlikely to be generalisable to all LMICs. The evidence of potential publication bias also indicates the need for greater awareness of the importance of publishing null findings. Recent evidence has indicated that scientists across disciplines hold a shared view that publishing null findings would be difficult/impossible [31]. It is possible that the LMICs missing in our review are the ones where the association between psychiatric disorders and suicide is weak or absent.

Our findings suggest that depression and mood disorders (23-27%) have the highest attributable risk for suicide. This PAF is lower or consistent with the point estimates for low levels of education in males (41%) and females (20%) from a review of evidence from primarily HIC studies [26]. Previous studies have estimated the PAF of socioeconomic and other social factors in LMICs, which highlight the importance of domestic abuse and economic influences [13, 32]. In a context of scarce economic resources, as is the case in many LMICs, the focus of prevention efforts needs to take into account where the expenditure of funds is likely to have the greatest effect. The estimation of PAFs are not routinely done in studies conducted in LMICs, and there have thus far not been any attempts to synthesis estimates for social factors.

Whilst this review focuses on estimating the PAF for psychiatric disorders, it is important to note that these disorders are unlikely to occur in isolation, especially in LMICs. Their development, maintenance or exacerbation are likely to be closely linked to social and structural issues that are prevalent in these countries. Issues such as poverty, unemployment, limited access to mental health support, and gender-based violence are all factors shown increase risk of suicide, with the risk further heightened when these factors intersect together.

## Strength and limitations

This is the first systematic approach to determine the impact of psychiatric disorders on suicide in the general population in LMICs including papers published in any language. There are, however, methodological limitations of this review which should be considered when interpreting the results. First, the search was conducted in three databases which may exclude some papers, particularly those from LMIC journals which are less likely to be indexed in these databases. To mitigate against this, we searched the references of all included papers and relevant reviews. Second, we did not include non-peer reviewed articles, and this would therefore exclude unpublished student dissertations which may have had additional data which would have been informative for this study. Last, we considered the presence of psychiatric diagnoses irrespective of time scale (i.e. lifetime or during a certain period before death), which means that the assumption of causality between psychiatric disorder and suicide may not hold.

## Conclusions

PAFs are able to help policy makers in decision making by ensuring that scarce resources are allocated in the most effective way for suicide prevention. There was considerable heterogeneity between study estimates included in this analysis, and an absence of data from the majority of LMICs.

## Supplementary Information

Below is the link to the electronic supplementary material.

Supplementary Material 1

Supplementary Material 2

Supplementary Material 3

## Data Availability

All data used in the manuscript are presented in a supplementary file.

## Abbreviations

LMIC: Low–and middle–income countries
PAF: Population attributable fraction
CI: Confidence interval
JBI: Joanna Briggs Institute
SCID: Structured Clinical Interview for DSM Disorders
