# Supplementary material for: Population attributable fractions of psychiatric disorders for suicide in low and middle-income countries: a systematic review and meta-analysis
Source: BMC Psychiatry. 2026 Jun 3;26:612. doi: 10.1186/s12888-026-08252-1 (PMC13466122; doi:10.1186/s12888-026-08252-1)
Supplement: Supplementary file 1 [file 12888_2026_8252_MOESM2_ESM.docx]

**Search Terms used – example Medline**

((mental and disorder) or (mental and illness) or (mental and disease) or (psychiatr* and disorder) or (psychiatr* and illness) or (psychiatr* And disease) or (psychiatr* and diagnosis) or (psychological and autopsy) ).ab OR ((mental and disorder) or (mental and illness) or (mental and disease) or (psychiatr* and disorder) or (psychiatr* and illness) or (psychiatr* And disease) or (psychiatr* and diagnosis) or (psychological and autopsy) ).ti

AND

(self?harm* or suicid*).ab OR (self?harm* or suicid*).ti

AND

(Afghanistan* or Albania* or Algeria* or Angola* or Argentina* or Armenia* or Azerbaijan* or Bangladesh* or Belarus* or Beliz* or Benin* or Bhutan* or Bolivia* or Bosnia* or Herzegovin* or Botswan* or Brazil* or Bulgaria* or Burkina* or Burundi* or Cabo Verde* or Cape Verde* or Cambodia* or Cameroon* or Central African or Chad* or China or Chinese or Colombia* or Comor* or Congo* or Costa Rica* or Cote d'Ivoir* or Ivory Coast or Cuba* or Djibouti* or Dominica* or Ecuador* or Egypt* or El Salvador* or Eritrea* or Ethiopia* or Fiji* or Gabon* or Gambia* or Georgia* or Ghana* or Grenad* or Guatemala* or Guinea* or Guyan* or Haiti* or Hondura* or Hungar* or India* or Indonesia* or Iran* or Iraq* or Jamaica* or Jordan* or Kazakhstan* or Kenya* or Kiribati* or Korea* or Kosov* or Kyrgyz Republic or Lao* or Leban* or Lesotho* or Liberia* or Libya* or Macedonia* or Madagascar* or Malawi* or Malaysia* or Maldiv* or Mali* or Marshall Island* or Mauritania* or Mauriti* or Mexic* or Micronesia* or Moldova* or Mongolia* or Montenegr* or Morocc* or Mozambi* or Myanma* or Burmese or Namibia* or Nepal* or Nicaragua* or Niger* or Nigeria* or Pakistan* or Palau* or Panama* or Papua New Guinea* or Paraguay* or Peru* or Philippines or Filipino or Romania* or Rwanda* or Samoa* or Sao Tome* or Senegal* or Serbia* or Seychell* or Sierra Leon* or Solomon Island* or Somalia* or South Africa* or Sudan* or Sri Lanka* or St Lucia* or St Vincent or Grenadines or Surinam* or Swazi* or Syria* or Tajikistan* or Tanzania* or Thai* or Timor* or Togo* or Tonga* or Tunisia* or Turk* or Turkmenistan* or Tuvalu* or Uganda* or Ukrain* or Uzbekistan* or Vanuatu* or Venezuela* or Vietnam* or West Bank or Gaza or Yemen* or Zambia* or Zimbabwe*).ab OR (Afghanistan* or Albania* or Algeria* or Angola* or Argentina* or Armenia* or Azerbaijan* or Bangladesh* or Belarus* or Beliz* or Benin* or Bhutan* or Bolivia* or Bosnia* or Herzegovin* or Botswan* or Brazil* or Bulgaria* or Burkina* or Burundi* or Cabo Verde* or Cape Verde* or Cambodia* or Cameroon* or Central African or Chad* or China or Chinese or Colombia* or Comor* or Congo* or Costa Rica* or Cote d'Ivoir* or Ivory Coast or Cuba* or Djibouti* or Dominica* or Ecuador* or Egypt* or El Salvador* or Eritrea* or Ethiopia* or Fiji* or Gabon* or Gambia* or Georgia* or Ghana* or Grenad* or Guatemala* or Guinea* or Guyan* or Haiti* or Hondura* or Hungar* or India* or Indonesia* or Iran* or Iraq* or Jamaica* or Jordan* or Kazakhstan* or Kenya* or Kiribati* or Korea* or Kosov* or Kyrgyz Republic or Lao* or Leban* or Lesotho* or Liberia* or Libya* or Macedonia* or Madagascar* or Malawi* or Malaysia* or Maldiv* or Mali* or Marshall Island* or Mauritania* or Mauriti* or Mexic* or Micronesia* or Moldova* or Mongolia* or Montenegr* or Morocc* or Mozambi* or Myanma* or Burmese or Namibia* or Nepal* or Nicaragua* or Niger* or Nigeria* or Pakistan* or Palau* or Panama* or Papua New Guinea* or Paraguay* or Peru* or Philippines or Filipino or Romania* or Rwanda* or Samoa* or Sao Tome* or Senegal* or Serbia* or Seychell* or Sierra Leon* or Solomon Island* or Somalia* or South Africa* or Sudan* or Sri Lanka* or St Lucia* or St Vincent or Grenadines or Surinam* or Swazi* or Syria* or Tajikistan* or Tanzania* or Thai* or Timor* or Togo* or Tonga* or Tunisia* or Turk* or Turkmenistan* or Tuvalu* or Uganda* or Ukrain* or Uzbekistan* or Vanuatu* or Venezuela* or Vietnam* or West Bank or Gaza or Yemen* or Zambia* or Zimbabwe*).ti
