# Supplementary material for: Population attributable fractions of psychiatric disorders for suicide in low and middle-income countries: a systematic review and meta-analysis
Source: BMC Psychiatry. 2026 Jun 3;26:612. doi: 10.1186/s12888-026-08252-1 (PMC13466122; doi:10.1186/s12888-026-08252-1)
Supplement: Supplementary file 2 [file 12888_2026_8252_MOESM3_ESM.docx]

Table S1: Population attributable fractions for exposure to psychiatric disorders and suicide

| Exposure | Mean proportion exposed | PAF (95% CI) |
| --- | --- | --- |
| Any psychiatric, axis 1 or SCID-I disorder | 14.2% | 67.3% (54.9%-77.4%) |
| Depression | 5.2% | 33.6% (19.0%-50.8%) |
| Mood disorders | 2.9% | 28.4% (12.7%-50.1%) |
| Substance use disorder | 6.1% | 15.8% (9.0%-24.7%) |
| Personality disorders | 2.2% | 10.1% (3.4%-22.4%) |
| Bipolar disorders | 0.9% | 7.6% (1.5%-25.0%) |
| Anxiety disorders | 4.1% | 7.1% (1.7%-16.5%) |
| Schizophrenia spectrum disorders | 0.6% | 5.7% (3.0%-10.2%) |
| Stress disorders | 1.7% | 4.1% (0.9%-10.6%) |
| Psychotic disorders | 0.1% | 1.1% (0.2%-4.3%) |
